# Systematic Analysis of the R2R3-MYB Family in *Camellia sinensis*: Evidence for Galloylated Catechins Biosynthesis Regulation

**DOI:** 10.3389/fpls.2021.782220

**Published:** 2022-01-03

**Authors:** Jingyi Li, Shaoqun Liu, Peifen Chen, Jiarong Cai, Song Tang, Wei Yang, Fanrong Cao, Peng Zheng, Binmei Sun

**Affiliations:** Key Laboratory of Biology and Germplasm Enhancement of Horticultural Crops in South China, Ministry of Agriculture and Rural Areas, College of Horticulture, South China Agricultural University, Guangzhou, China

**Keywords:** *Camellia sinensis*, galloylated catechins, R2R3-MYB, transcriptional regulation, catechin biosynthesis

## Abstract

The R2R3-MYB transcription factor (TF) family regulates metabolism of phenylpropanoids in various plant lineages. Species-expanded or specific MYB TFs may regulate species-specific metabolite biosynthesis including phenylpropanoid-derived bioactive products. *Camellia sinensis* produces an abundance of specialized metabolites, which makes it an excellent model for digging into the genetic regulation of plant-specific metabolite biosynthesis. The most abundant health-promoting metabolites in tea are galloylated catechins, and the most bioactive of the galloylated catechins, epigallocatechin gallate (EGCG), is specifically relative abundant in *C. sinensis*. However, the transcriptional regulation of galloylated catechin biosynthesis remains elusive. This study mined the R2R3-MYB TFs associated with galloylated catechin biosynthesis in *C. sinensis*. A total of 118 R2R3-MYB proteins, classified into 38 subgroups, were identified. R2R3-MYB subgroups specific to or expanded in *C. sinensis* were hypothesized to be essential to evolutionary diversification of tea-specialized metabolites. Notably, nine of these *R2R3-MYB* genes were expressed preferentially in apical buds (ABs) and young leaves, exactly where galloylated catechins accumulate. Three putative *R2R3-MYB* genes displayed strong correlation with key galloylated catechin biosynthesis genes, suggesting a role in regulating biosynthesis of epicatechin gallate (ECG) and EGCG. Overall, this study paves the way to reveal the transcriptional regulation of galloylated catechins in *C. sinensis*.

## Introduction

Tea from *Camellia sinensis*, along with coffee and cocoa, is one of the world’s three major non-alcoholic beverages. Worldwide, approximately two billion cups of tea are consumed daily (Drew, 2019; Yamashita et al., 2020). Tea production has amplified at an average annual rate of 3.35% in the last 5 years; by 2019, worldwide tea production reached 6.49 million tons on 5.07 million hectares (Food and Agriculture Organization of the United Nations statistics^1^). Tea was used first as a food in ancient China, then it served as a medicine to prevent and cure common diseases before developing into a popular beverage (Mondal et al., 2004; Abbas et al., 2017). Nowadays, tea exhibits great commercial potential and has become a vital industry due to its health promoting properties and attractive distinct flavors (Chen et al., 2009).

The tea plant (*C. sinensis*) is rich in characteristic metabolites, such as polyphenols, amino acids, caffeine, and terpenes, that significantly contribute to its pleasant flavors and industrial and medical value (Wei et al., 2018). Catechins are the principal health-promoting bioactive compounds of tea. Catechins constitute 12–24% of the dry weight of young leaves, and account for more than 70% of the total polyphenols (Yang et al., 2012). Catechins in tea consist of a mixture of catechin (C), epicatechin (EC), gallocatechin (GC), epigallocatechin (EGC), catechin gallate (CG), epicatechin gallate (ECG), gallocatechin gallate (GCG), and epigallocatechin gallate (EGCG) (Asakawa et al., 2013). Among them, galloylated catechins ECG and EGCG are relatively abundant in tea plants and account for more than 80% of total catechins (Kim et al., 2004; Liu et al., 2012). EGCG, the most specific accumulated metabolite that quantitatively differentiated from the other plants, is the major bioactive component conferring the many health benefits of tea – it is anti-carcinogenic (Ahmad et al., 2000), anti-oxidative (Heim et al., 2002), anti-bacterial and anti-inflammatory (Taguri et al., 2004), and it prevents cardiovascular and cerebrovascular diseases (Yu X. et al., 2020). In addition, EGCG is widely used in food production on account of its strong antioxidative capacity (Nikoo et al., 2018).

To date, based on biochemical, physiological, and genetic research, the biosynthesis pathway of catechins has become clear (Yang et al., 2012; Wei et al., 2018; Yu et al., 2021). Catechins are derived from the phenylpropanoid pathway and principally accumulate in apical shoots and young leaves. Galloylated catechin content is mainly regulated at the transcriptional level by catechin biosynthesis genes *dihydroflavonol reductase* (*DFR*), *anthocyanidin reductase* (*ANR*), *leucoanthocyanidin reductase* (*LAR*), and *serine carboxypeptidase-like acyltransferases* (*SCPLs*) (Punyasiri et al., 2004; Singh et al., 2008; Eungwanichayapant and Popluechai, 2009; Ashihara et al., 2010; Wei et al., 2018). However, few studies have focused on the network(s) that regulate catechin biosynthesis, especially galloylated catechins.

R2R3-MYB transcription factors (TFs) comprise the largest family of TFs in advanced plants (Ambawat et al., 2013). In addition to possessing two imperfect MYB repeats (R2 and R3), the R2R3-MYB TFs maintain a highly conserved N-terminal MYB DNA-binding domain and an activated or repressed C-terminal domain (Lipsick, 1996; Martin and Paz-Ares, 1997; Kranz et al., 1998; Jin and Martin, 1999; Jiang et al., 2004; Dubos et al., 2010). The R2R3-MYB family is widely involved in plant growth and development, primary and secondary metabolism, hormone signal transduction, cellular proliferation, and apoptosis, as well as disease and abiotic stress response (Martin and Paz-Ares, 1997; Li M. et al., 2017). Notably, the R2R3-MYB family plays an important role in positively or negatively regulating the biosynthesis of specialized metabolites, such as flavonoids (Mehrtens et al., 2005; Hichri et al., 2011), anthocyanin (Li W. et al., 2017; Li et al., 2020; Yu M. et al., 2020), and lignin (Goicoechea et al., 2005; Bedon et al., 2007). Studies have found that new R2R3-MYB TFs emerged through species-specific duplication events (Soler et al., 2015). Species-specific evolved or expanded R2R3-MYB membership seems to confer functional diversification to organisms (Zhang et al., 2000). For example, the ancestral R2R3-MYB anthocyanin master regulator expanded into several homologous clusters within the grape (*Vitis* spp.) and maize (*Zea mays*) genomes, and differential expression of duplicated genes resulted in control of anthocyanin biosynthesis in different tissues (Zhang et al., 2000; Jiu et al., 2021). Some species-specific and expanded *R2R3-MYB* TFs govern specialized metabolite biosynthesis within lineages (Zhu et al., 2019). In *Capsicum*, five Solanaceae-specific MYB TF tandem genes duplicated in the *Cap1/Pun3* locus. *Capsicum* species have evolved placenta-specific expression of *MYB31*, which directly activates expression of capsaicinoid biosynthetic genes and results in production of genus-specialized metabolites.

In *C. sinensis*, some R2R3-MYB TFs have a demonstrated role in regulation of phenylpropanoid biosynthesis in *C. sinensis*, such as *CsAN1*, *CsMYB5a*, *CsMYB6A*, and *CsMYB75* that regulate anthocyanin pigments and *CsMYB5e* and *CsMYB5b* that involve in proanthocyanidin biosynthesis in *C. sinensis* tissues (Sun et al., 2016; He et al., 2018; Jiang et al., 2018; Wei et al., 2019; Wang et al., 2019, 2020). Recently, Chen et al. (2021) analyzed the genome-wide R2R3-MYB superfamily and found that some *CsR2R3-MYB* genes in response to drought, cold, gibberellic acid (GA), and abscisic acid (ABA) treatments and a key *CsR2R3-MYB* gene regulated some main anthocyanin components. However, the *C. sinensis* specific and expanded R2R3-MYB TFs that are potential candidate regulators of galloylated catechins biosynthesis have still not been identified.

Because of the importance of galloylated catechins in *C. sinensis*, this study focused on the specifically expanded *CsR2R3-MYB* genes and the high content of EGCG in *C. sinensis.* Differentiating from previous works, we adapted a more general genome-wide analysis method of the R2R3-MYB superfamily according to *Arabidopsis thaliana*, which greatly help to find out the specifically expanded *CsR2R3-MYB* genes and the high content of EGCG in *C. sinensis* (Soler et al., 2015; Li M. et al., 2017; Chen et al., 2021). The gene structure, conserved motifs and transcript patterns were also analyzed. Notably, three putative *R2R3-MYB* genes specifically expanded in tea plants exhibited strong correlation with key galloylated catechin biosynthesis genes and galloylated catechins content, suggesting a possible role in the regulating network of the biosynthesis of ECG and EGCG. This study proposed a new perspective of the possible transcriptional regulation mechanism with regard to the high accumulation of galloylated catechins in *C. sinensis*.

## Materials and Methods

### Plant Materials

The “Lingtoudancong” variety of *C. sinensis* was grown at South Agricultural University in Guangzhou, China. Apical buds (ABs), first leaves, second leaves, mature leaves, old leaves, stems, and roots of three plants were sampled as one biological replicates in spring of 2021. Three biological replicates of different tissues were immediately frozen in liquid nitrogen and stored at −80°C.

### Phylogenetic Analysis

*Camellia sinensis* MYB protein sequences were retrieved from the Tea Plant Information Archive database.^2^ In total, 222 MYBs and MYB-related genes were predicted in the ‘‘Shuchazao’’ genome, and they were all submitted to Pfam website^3^ to search for the two consecutive and conserved repeats of the MYB domain. However, only 118 of the MYBs TFs fit this characteristic, which were different from previous studies (Chen et al., 2021). The R2R3-MYB protein sequences of *A. thaliana* were obtained from the Arabidopsis Information Resource Archive database.^4^ The number of R2R3-MYB gene models identified by our methodology in the genome of *A. thaliana* (126) was the same as described in the literature (Dubos et al., 2010). The homologous genes of kiwifruit, coffee, cacao and grape were retrieved from the Plant Transcription Factor Database^5^ by performing a reverse BLAST search. All the R2R3-MYB sequences were aligned using ClustalX, and a neighbor-joining phylogenetic tree was constructed with 1,000 bootstrap replicates utilizing MEGAX (Kumar et al., 2016). A pairwise deletion method was chosen to dispose of the positions containing gaps or missing data in the sequences, and the delay divergent cut-off value was set to 30.

### Synteny Analysis

Toolkits of TBtools software were used to carry out the synteny analysis and K_a_/K_s_ value calculation (Chen et al., 2020). Syntenic blocks and distinct duplication events were identified by One Step MCScanX and the synteny relationships of the orthologous *R2R3-MYB* genes between the *C. sinensis* and the other selected four representative plant species were displayed by Dual Synteny Plot. Besides, the K_a_ and K_s_ values of each duplicated *MYB* gene pair were counted by Simple K_a_/K_s_ Calculator.

### Conserved Motif and Myb-Binding Sites Analysis

Functional motifs and conserved domains were identified with The MEME Suite tool^6^ using the following parameters: site distribution, zero-or-one-site-per-sequence (ZOOPS) model; maximum number of motifs: 20; minimum motif width: 6; maximum motif width: 50; minimum number of sites per motif: 2; and maximum number of sites per motif: 118 (Bailey et al., 2009; Chen et al., 2021). The sequence logos of R2 and R3 repeats of the R2R3-MYB proteins were based on multiple sequence alignments and were visualized with WebLogo Version 2.8.2.^7^ All obtained motifs were constructed and visualized using the Gene Structure View (Advanced) of the TBtools software. The Myb-binding sites were predicted in the 2000-bp upstream regions of the catechin biosynthesis downstream genes with PlantCARE online website^8^.

### RNA-Seq Expression Analysis

The RNA-seq data were downloaded from TPIA for transcript abundance analyses. The expression levels of the candidate *R2R3-MYB* TFs and catechin biosynthesis genes from different tissues of *C. sinensis* were used to generate a heatmap with TBtools software using the normalized method.

### RNA Extraction and Quantitative Real-Time Polymerase Chain Reaction

Total RNA of different tissues of “Lingtoudancong” was extracted utilizing a Magen HiPure Plant RNA Mini kit B (R4151, Magen, China) according the manufacturer’s instructions. First-strand cDNA was synthesized using a HiScript III RT 1st Strand cDNA Synthesis kit (R323-01, Vazyme, China) in a reaction volume of 20 μL. Quantitative real-time polymerase chain reaction (qRT-PCR) was performed in a Bio-Rad CFX384 Touch™ system. Each 10 μL reaction mixture was comprised of 4.4 μL qPCR SYBR Green Master Mix (Yeasen, China), 4.4 μL double distilled water, 0.2 μL of each primer (10 μmol/μL) and 1 μL of cDNA template. The reaction program was as follows: 95°C for 5 min; then 39 cycles at 95°C for 5 s and 60°C for 30 s. A melting-curve analysis was carried out at 95°C for 5 s, which was followed by a temperature increase from 60 to 95°C. All qRT-PCR reactions were carried out in independent triplicate and *Actin* (*TEA019484.1)* was used as the housekeeping gene (Guo et al., 2019; Zhou et al., 2019). The relative expression of each gene was calculated with the 2^–ΔΔCt^ method as introduced in the previous research and the values were the means ± SDs (Livak and Schmittgen, 2001). The qRT-PCR primers were designed with the qPrimerDB-qPCR Primer Database.^9^ Sequences of the primers are listed in Supplementary Table 1.

### Quantification of Catechin Contents

Reference standards of C, EC, EGC, ECG, and EGCG were purchased from Shanghai Yuanye Bio-Technology Co., Ltd. (Shanghai, China). ABs, first leaves, second leaves, mature leaves, old leaves, stems and roots of “Lingtoudancong” were ground into fine powders and freeze-dried. Approximately 0.2 g of each sample powder was extracted with 8 mL of 70% methyl alcohol (diluted with ultrapure water). After ultrasonic extraction for 30 min, the supernatant was collected by centrifugation. One milliliter of the liquid supernatant was filtered through a 0.22 μm Millipore membrane. The extracts of each sample were independently injected into three XSelect HSS C18 SB columns (4.6 × 250 mm, 5 μm, Waters Technologies, United States) for three independent replicates. The catechin monomers were separated using 0.1% aqueous formic acid (A) and 100% acetonitrile (B) as mobile phases on a Waters Alliance Series HPLC system (Waters Technologies, United States). Detection was performed at 280 nm. The data were presented as the mean ± SD (*n* = 3).

### Correlation Analysis of Gene Expression and Metabolite Accumulation

The correlation analysis among TFs, catechin biosynthesis genes, and catechin monomer contents was performed *via* Pearson’s correlation coefficients. The R software was adopted to visualize the relationship directly. A correlation coefficient of >0.5 was considered to be a positively associated pair, and *R* < −0.5 was thought of as a negative correlation. In the diagram, blue represents a positive correlation, and red represents a negative correlation.

## Results

### Comparative Phylogenetic Analysis of the R2R3-MYB Families in *Camellia sinensis* and *Arabidopsis thaliana*

**FIGURE 1:**
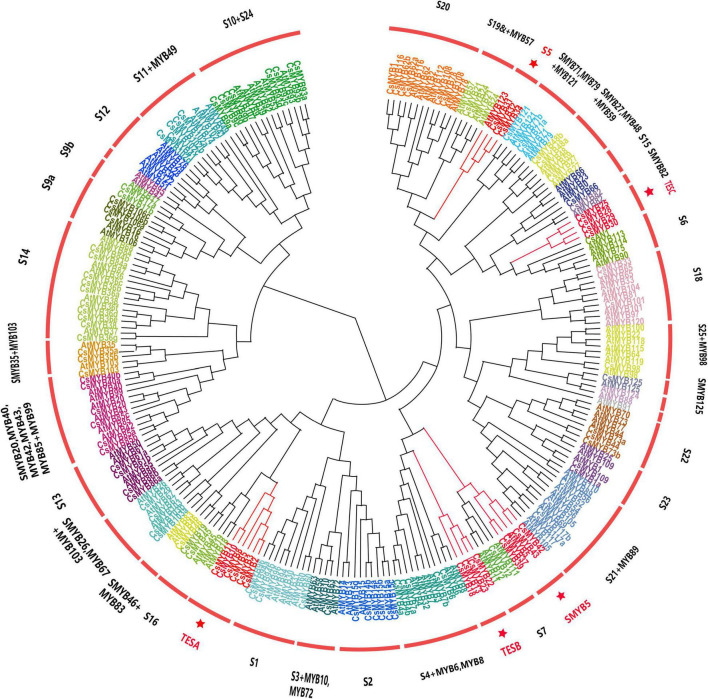
Phylogenetic analysis of the R2R3-MYB families in *C. sinensis* and *A. thaliana*. A neighbor-joining phylogenetic tree was constructed from 244 protein sequences including all R2R3-MYB proteins from *C. sinensis* (118) and *A. thaliana* (126). Subgroups within each clade were given a different color; meanwhile, the same color indicates the genes are in the same subgroup. Subgroup short names are included next to each clade to simplify nomenclature. Subgroups that evolved and expanded exclusively in the *C. sinensis* genome are highlighted in red and marked with a red star. TESA, tea expanded subgroup A; TESB, tea expanded subgroup B; TESC, tea expanded subgroup C; SMYB5, subgroup MYB5; S5, subgroup 5.

A total of 118 *R2R3-MYB* genes were identified in the *C. sinensis* genome after manual curation and exclusion of alternative transcripts. All identified *R2R3-MYB* genes from *C. sinensis* (118) were aligned with those of *A. thaliana* (126), and their evolutionary history was inferred by constructing a neighbor-joining phylogenetic tree (Figure 1). The 118 *R2R3-MYB* genes of *C. sinensis* were named in light of the systematic naming rules of *A. thaliana*, except for *CsMYB1*, *CsMYB4a*, and *CsAN1*, which had been functionally characterized previously (Supplementary Table 3; Yang et al., 2012; Sun et al., 2016; Li M. et al., 2017). In addition, as it is believed that genes which clustered together were considered to be in the same subgroup, and *A. thaliana* is, by far, the species for which the *R2R3-MYB* genes have been most extensively investigated, the 38 subgroups were classified by taking into account the topology of the tree and the bootstrap values (Figure 1 and Supplementary Table 3) and were named according to the classification of *A. thaliana*. For new subgroups not previously proposed in *A. thaliana*, the subgroup was named after the known functionally characterized *A. thaliana* member.

The majority of subgroups contained members from both species. However, S12 was an *Arabidopsis*-specific subgroup, containing only *R2R3-MYB* members from *A. thaliana*. The members of subgroup S12, *AtMYB28*, *AtMYB29*, *AtMYB76*, *AtMYB34*, and *AtMYB5*1 regulate glucosinolate biosynthesis, a metabolite exclusive to the Brassicaceae family (Gigolashvili et al., 2007; Matus et al., 2008). In contrast, three subgroups contained *R2R3-MYB* TFs that were present only in *C. sinensis* without any homologs in *A. thaliana*. Therefore, their names we designated as Tea Expanded Subgroup A (TESA), Tea Expanded Subgroup B (TESB), and Tea Expanded Subgroup C (TESC). Remarkably, SMYB5 and S5 subgroups were comprised of more tea plant *R2R3-MYB* members than *A. thaliana* members. For example, subgroup S5 had three *C. sinensis* members (*CsMYB31*, *CsMYB32*, and *CsMYB34*), while *A. thaliana* contributed only one member, *AtMYB123/TT2.* Similarly, the SMYB5 subgroup had four members from *C. sinensis* (*CsMYB37*, *CsMYB39*, *CsMYB42*, and *CsMYB43*), but just one member (*AtMYB5*) from *A. thaliana.* In *Arabidopsis*, the members of SMYB5 and S5 subgroups are involved in the phenylpropanoid pathway. *AtMYB123* controls the biosynthesis of proanthocyanidins (PAs) in the seed coat and *AtMYB5* was partially redundant with *AtMYB123* (Gonzalez et al., 2009). The expansion of SMYB5 and S5 subgroups in *C. sinensis* suggests that diversified regulation of polyphenols emerged during the speciation of *C. sinensis*.

Overall, the phylogenetic analysis results highlighted five special subgroups TESA, TESB, TESC, S5, and SMYB5, which expanded in or were exclusively present in *C. sinensis*. Among these subgroups, a total of 21 R2R3-MYB TFs [TESA (6), TESB (4), TESC (4), S5 (3) and SMYB5 (4)] attracted our attention and were selected for further analysis as potential candidates involved metabolic processes specific to *C. sinensis*.

### Functions Inferred Through Phylogenetic Analysis of the Candidate Subgroups in Six Plant Species

To evaluate the 21 *R2R3-MYB* genes of the five tea specifically expanded subgroups TESA, TESB, TESC, S5, and SMYB5, the homologous *R2R3-MYB* genes from kiwifruit, coffee, cacao, and grape were used to construct phylogenetic tree (Supplementary Figure 1). The number of *R2R3-MYB* TFs presented a trend of expansion in *C. sinensis* (21) similar to coffee (20), but greater than cocoa (11), kiwifruit (11), and grape (18). We surmised that the *C. sinensis* expanded *R2R3-MYB* function may have occurred through divergent evolution during speciation. For example, subgroup TESA was expanded in tea (6) relative to coffee (3), cocoa (5), kiwifruit (5), and grape (1). In the TESB subgroup, coffee (4), grape (4), and kiwifruit (3) had comparable numbers of members with *C. sinensis* (4), but only two homologous genes were found in cocoa. Subgroup TESC contained homologous genes in all species except for kiwifruit. Remarkably, no isogenous genes of TESA, TESB, or TESC subgroups were present in *A. thaliana*, indicating that these *R2R3-MYB*s evolved only in tea plant and probably have novel functions.

To uncover the roles these *R2R3-MYB* genes serve, we searched for the functional characteristics of the selected *R2R3-MYB* genes from four close relative species. Only a few homologous genes (*GSVIVT01026868001*, *Achn38246*, and *Achn172901* from the TESA subgroup; *Achn143561*, *Achn322351*, and *GSVIVT0103866001* from the TESB subgroup; and *Thecc1EG029126t1*, *GSVIVT01016765001*, and *GSVIVT01035467001* from TESC subgroup) have been experimentally verified. In the TESA subgroup, *GSVIVT01026868001* played an inhibitory role in flower development (Velasco et al., 2007), while both *Achn38246* and *Achn172901* acted as transcriptional activators involved in cold stress response (Park et al., 2010; Savage et al., 2013). Homologous genes in the TESB subgroup, *Achn143561*, *Achn322351*, and *GSVIVT0103866001*, performed a similar role in regulating plant protection against UV stress (Schenke et al., 2014). The paralogous genes of subgroup TESC (*Thecc1EG029126t1*, *GSVIVT01016765001*, and *GSVIVT01035467001*) mainly regulated plant epidermal cell fate (Savage et al., 2013; Cheng et al., 2014). Above all, we ventured that the function of the TESA, TESB, and TESC subgroups in *C. sinensis* might associate with responses to biotic and abiotic stress along with influencing certain developmental processes.

For detecting the driving force for evolution of the *C. sinensis R2R3-MYB* gene family, non-synonymous and synonymous substitution ratio (K_*a*_ and K_*s*_) analyses were performed among the whole *CsR2R3-MYB* genes (Supplementary Table 4). Additionally, collinearity analysis among *C. sinensis* and the four selected plant lineages was carried out to explore the evolutionary mechanisms of the *C. sinensis* 2R-MYB family (Supplementary Figure 2 and Supplementary Tables 5–8).

## Conserved Motif Analysis of *R2R3-MYB*s

The R2 and R3 MYB domains of the 118 *C. sinensis R2R3-MYB* TFs were analyzed (Supplementary Figure 3). The R2 and R3 domains contain a set of characteristic amino acids, which include the highly conserved and evenly distributed tryptophan residues (Trp, W) known to be critical for sequence-specific binding of DNA (Stracke et al., 2001; Cao et al., 2013), demonstrating that the R2 and R3 MYB repeats of the MYB DNA-binding domain are highly conserved in *C. sinensis*, consistent with previous findings of the counterpart genes in other plant lineages (Wilkins et al., 2009; Du et al., 2012; Li et al., 2016). Most of the conserved residues were situated between the second and third conserved W residues in each MYB repeat, elucidating that the first area of them was less conserved than the other two.

Conserved amino acid motifs represent functional areas that are maintained during evolution. The conserved motifs within the 118 R2R3-MYB sequences were analyzed and aligned using MEME Suite. A total of 20 conserved motifs were identified in the R2R3-MYB family (Supplementary Figure 4). Six of these, motifs 1–6, were present in all R2R3-MYB members except for CsMYB1a and thus were designated as “general motifs”; the rest of the motifs (motifs 7–20) were considered to be “specific motifs,” since they were present in only one or several R2R3-MYB members. For instance, motifs 16 and 9 were unique to CsMYB1a and CsMYB117a; meanwhile, motifs 15 and 19 were contained only in three genes. Overall, the members clustering to the same clade harbored similar motif patterns.

### Expression Patterns of the R2R3-MYB Family

**FIGURE 2:**
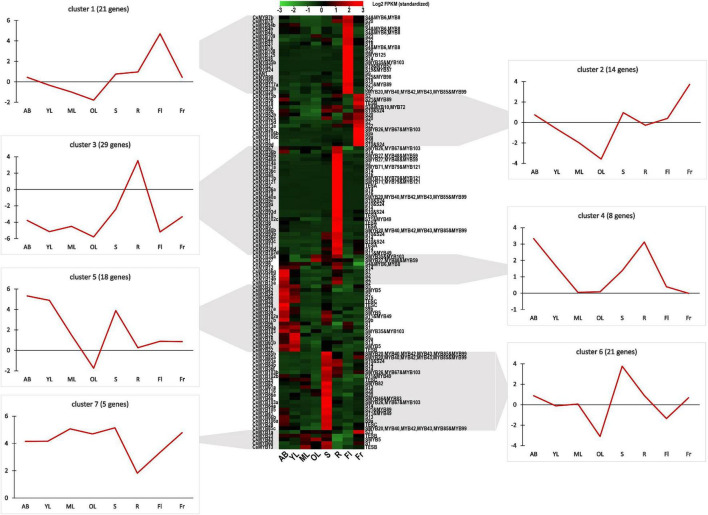
Heatmap of the 118 *CsR2R3-MYB* genes transcribed in the tissues of *C. sinensis*. The 118 *CsR2R3-MYB* genes clustered into seven expression groups, based on their tissue-specific expression. The gene name is indicated on the left of the heatmap, and the short name of the phylogenetic subgroup is on the right. Transcript abundance is expressed in standardized log2 fragments per kilobase of exon per million fragments mapped (FPKM) values. Next to each RNA-seq-based cluster, there is a graph with the mean transcript abundance for the entire cluster in each tissue. AB, apical bud; YL, young leaf; ML, mature leaf; OL, old leaf; S, stem; R, root; Fl, flower; Fr, fruit. Data were obtained from the Tea Plant Information Archive (http://tpia.teaplant.org/).

The expression patterns of 118 genes encoding *R2R3-MYB* TFs were analyzed in different tissues. No transcripts were detected for *CsMYB101* (*TEA028392.1*) and *CsMYB117b* (*TEA002233.1*), suggesting they are pseudogenes in *C. sinensis*. The genes were classified into seven expression clusters, based on their distinct transcript patterns in various tissues and organs (Figure 2). The 21 genes in RNA-seq-based cluster 1 were expressed mainly in flowers; genes in cluster 2 (14) were predominantly expressed in fruits; genes in cluster 3 (29) were mainly expressed in tender roots; genes in cluster 4 (8) were expressed at comparable levels in both ABs and tender roots; cluster 5 genes (18) were mainly present in apical shoots and young leaves; while cluster 6 genes (21) were mainly expressed in stems and finally, cluster 7 genes (5) were equally expressed in mature leaves, old leaves, and stems. Normally, genes within the same phylogenetic subgroup exhibit distinct transcript profiles (Dubos et al., 2010). Such was the case for subgroup S14: the members in this subgroup were detected in RNA-seq-based clusters 1, 3, and 6. In *A. thaliana*, members of S14 were generally related to axillary bud formation and cell differentiation. In some cases, however, genes belonging to the same subgroups might also have similar transcription profiles in the same tissue, organ or cell type. Such was the case with the TESA subgroup; all were present in RNA-seq-based cluster 3. Likewise, all members of subgroup S5 gathered in cluster 5.

Previous reports have pointed out that most of the genes involved in flavonoid formation and catechin biosynthesis were preferentially expressed in ABs and young leaves, where most galloylated catechins accumulate (Wei et al., 2018). Accordingly, *R2R3-MYB*s in RNA-seq-based cluster 5, whose expression level was highest in these two tender tissues, are the most likely candidates regulating catechin biosynthesis. As is shown in Figure 2, almost half of the genes found in cluster 5 (9) belonged to the subgroups that specifically evolved in or expanded in *C. sinensis*, subgroups S5, SMYB5, TESB, and TESC. It is worth noting that genes of subgroup S5 and SMYB5 were confirmed to be involved in flavonoid formation in *A. thaliana* (Nesi et al., 2001; Stracke et al., 2001), whereas subgroup TESB was inferred to be relevant to UV protection and subgroup TESC to control plant epidermal cell fate specification.

### *In silico* Analysis of R2R3-MYB Expression and Catechin Accumulation

**FIGURE 3:**
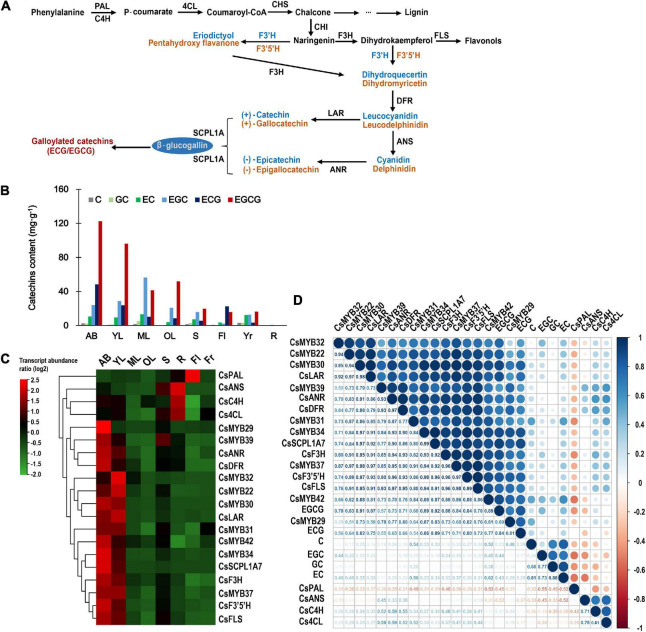
Expression pattern analysis and correlation analysis of tea-specific or tea-expanded *CsR2R3-MYB*s. **(A)** The biosynthetic pathway of catechins. *CHS*, *CHI*, *F3H*, *F3′H*, *F3′5′H*, *DFR*, *ANS*, *LAR*, *ANR*, and *SCPL1A* represent genes encoding chalcone synthase, chalcone isomerase, flavanone 3-hydroxylase, flavonoid 3′-hydroxylase, flavonoid 3′,5′-hydroxylase, dihydroflavonol 4-reductase, anthocyanidin synthase, leucoanthocyanidin reductase, anthocyanidin reductase, and type 1A serine carboxypeptidase-like acyltransferases, respectively. **(B)** The contents of six catechin monomers in eight tissues. **(C)** Heatmap of RNA-seq transcript abundance patterns of the 20 *CsR2R3-MYB* genes from the *C. sinensis* genome in eight different tissues. AB, apical bud; YL, young leaf; ML, mature leaf; OL, old leaf; S, stem; R, root; Fl, flower; Fr, fruit. **(D)** Correlative analysis of *CsR2R3-MYB* genes, structural genes, and catechins accumulation patterns in eight representative tissues of *C. sinensis* plants. *R* > 0.5 indicates a positive correlation; *R* < –0.5 indicates a negative correlation. Data were obtained from the Tea Plant Information Archive (http://tpia.teaplant.org/).

Catechins are the major type of polyphenols in the tea plant, comprising up to 70% of the polyphenols deriving from the phenylpropanoid pathway (Figure 3A). Figure 3B shows that catechin contents, especially the contents of the galloylated catechins EGCG and ECG, are significantly higher in ABs and young leaves than in other tissues. Therefore, for understanding the possible correlation between the galloylated catechins and the *CsR2R3-MYB*s that specifically evolved or expanded in *C. sinensis*, we focused on the nine *R2R3-MYB*s grouped in cluster 5 (*CsMYB22*, *CsMYB29*, *CsMYB30*, *CsMYB31*, *CsMYB32*, *CsMYB34*, *CsMYB37*, *CsMYB39*, and *CsMYB42*) because they were preferentially expressed in ABs and young leaves.

Further, the transcript abundance of the nine potential *R2R3-MYB* TFs and the catechin biosynthesis genes were investigated in different tissues. The results clearly showed that the genes in the catechin biosynthesis pathway display tissue-specific expression patterns. The genes downstream of catechin biosynthesis (*CsSCPL1A7*, *CsANR*, *CsLAR, CsDFR, CsFLS*, *CsF3H*, and *CsF3′5′H)* were highly expressed in ABs and young leaves, whereas the upstream genes (*CsPAL*, *Cs4CL*, and *CsC4H*) were highly expressed in root and flower (Figure 3C). The promotors of the catechin biosynthesis downstream genes (*CsSCPL1A7*, *CsANR*, *CsLAR, CsDFR, CsF3H*, and *CsF3′5′H*) have several Myb-binding domains (Supplementary Figure 5). Interestingly, the nine candidate *R2R3-MYB* TFs showed preferential expression in ABs and young leaves, which was consistent with the expression pattern of the downstream catechin biosynthetic genes, indicating that those *R2R3-MYB*s have relevance to catechin biosynthesis.

To identify the relationship between transcript abundance and catechin contents, a comprehensive gene-to-metabolite correlation analysis was conducted. As shown in Figure 3D, the three genes in the catechin biosynthesis pathway (*CsPAL*, *CsC4H*, and *Cs4CL*) that were not tender parts-specific indeed showed a low correlation or negatively correlated to catechin content. Comparatively, the expression level of the nine candidate *R2R3-MYB* TFs was positively correlated to the transcript abundance patterns of the catechin biosynthesis pathway downstream genes (*CsSCPL1A7*, *CsANR*, *CsLAR, CsDFR, CsFLS*, *CsF3H*, and *CsF3′5′H)* and correlated with the contents of EGCG and ECG, with inter-gene-to-metabolite Pearson’s correlation coefficients over 0.55. *CsMYB30*, *CsMYB34*, *CsMYB37*, and *CsMYB42* exhibited good performance compared with the others, each having a correlation coefficient exceeding 0.75, indicating an extremely strong correlation. Notably, *CsMYB34* had the strongest correlation with the catechin biosynthesis pathway downstream genes (>0.9), especially with *CsSCPL1A7* (0.99). Besides that, the coefficients between *CsMYB34* and EGCG and ECG contents were 0.89 and 0.86, respectively.

### Validation of the Correlation Between *R2R3-MYB* Transcription Factors and Catechins

**FIGURE 4:**
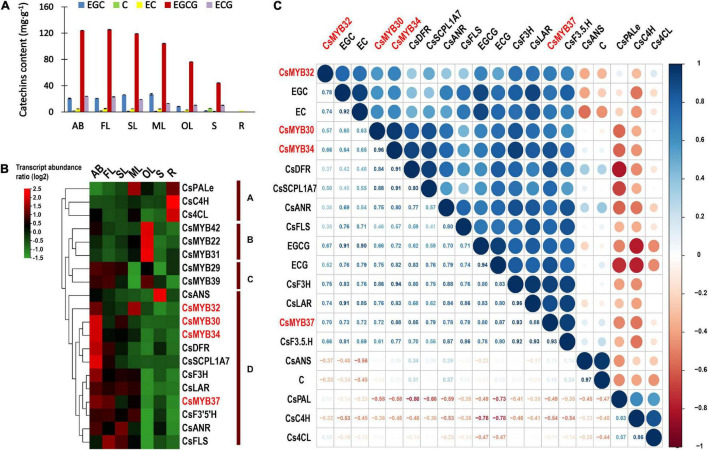
Expression profiling validation and correlation analysis. **(A)** The contents of six catechin monomers in seven tissues as measured with an HPLC system. **(B)** Heatmap of qRT-PCR transcript abundance patterns in seven different tissues. **(C)** Correlative analysis of four potential *CsR2R3-MYB* TFs, catechin biosynthesis genes and catechin accumulation patterns in different tissues of tea plants. *R* > 0.5, positive correlation; *R* < –0.5, negative correlation. AB, apical bud; YL, young leaf; SL, second leaf; ML, mature leaf; OL, old leaf; S, stem; R, root.

To validate the relationship between *R2R3-MYB* TFs and catechins, HPLC and qRT-PCR assays were carried out. Different tissues of the tea plant were tested for the contents of different catechin monomers *via* HPLC (Figure 4A and Supplementary Table 9). As shown in Figure 4A, there was a high level of EGCG in all tested tissues compared with the other catechin monomers, reaching the highest level in ABs and young leaves (FL and SL). Additionally, the expression patterns of the nine specially evolved and expanded candidate *CsR2R3-MYB*s and catechin biosynthesis genes in different tissues were verified by qRT-PCR. According to the relative expression patterns, there were four distinct clusters (clusters A–D) (Figure 4B). The genes upstream of catechin biosynthesis (*CsPAL*, *CsC4H*, and *Cs4CL*) grouped in cluster A and were highly expressed in roots, consistent with the absence of catechins in roots (Figure 3C). *CsMYB22*, *CsMYB31* and *CsMYB42* (cluster B) had distinctly high expression in old leaves, and were not AB- or young leaf-specific. Remarkably, *CsMYB30*, *CsMYB32*, *CsMYB34*, and *CsMYB37*, which clustered with critical genes downstream of catechins biosynthesis (cluster D) were highly expressed in ABs and first leaves, where EGCG and ECG accumulated (Figure 4A), preliminarily validating the intimate correlation between *CsMYB30*, *CsMYB32*, *CsMYB34*, *CsMYB37*, and catechin biosynthesis.

For further confirmation, gene-to-metabolite correlation analysis of *CsMYB30*, *CsMYB32*, *CsMYB34*, *CsMYB37*, key genes of the catechin biosynthesis pathway, and the accumulation of catechins was conducted (Figure 4C). The results confirmed the extremely low correlation between four of the candidate TFs and the genes upstream of catechin biosynthesis *CsPAL*, *CsC4H*, and *Cs4CL*. In contrast, *CsMYB30*, *CsMYB34*, and *CsMYB37* were strongly associated with most of the major genes downstream of the galloylated catechin biosynthesis pathway (*CsSCPL1A7*, *CsANR, CsLAR*, and *CsDFR*) as well as ECG and EGCG contents (>0.7). Particularly, *CsMYB37* showed the highest correlation level with the highest inter-correlation coefficients within the four major functional genes *CsSCPL1A7*, *CsANR*, *CsLAR, CsDFR* and the contents of EGCG and ECG reaching to 0.79, 0.78, 0.88, 0.85, 0.87 and 0.8, respectively. However, *CsMYB30*, *CsMYB34*, and *CsMYB37* were less related to the other downstream catechin biosynthesis genes (*CsFLS*, *CsF3H*, and *CsF3′5′H*) according to their lower Pearson’s correlation coefficients. Consistent with the *in silico* results in Figure 3D, the correlation among *CsMYB32*, the galloylated catechin contents and the biosynthesis genes was weaker than the other three *R2R3-MYB*s. In more detail, it had a relatively low correlation coefficient with ECG (0.52) and EGCG (0.67), and a relatively low correlation coefficient with genes *CsSCPL1A, CsANR, CsLAR*, and *CsDFR* (0.5, 0.36, 0.74 and 0.37, respectively).

The results provide convincing clues that *CsMYB30*, *CsMYB34*, and *CsMYB37* might be the key TFs regulating galloylated catechins biosynthesis for the abundance of EGCG in *C. sinensis*.

## Discussion

Plants are rich in metabolites that allow them to adapt to the environment and resist biotic and abiotic stress (Howe and Jander, 2008). These metabolites are widely used as natural products for treating human diseases and are valuable raw materials for modern industry (Plomion et al., 2001; Howe and Jander, 2008; Guo et al., 2018; Fang et al., 2019). *C. sinensis* is an advantageous model system to dig into plant-specific metabolite biosynthesis and genetic regulation with rich and differential metabolite profiles. Its metabolites, such as flavonoids, caffeine, and volatile terpenes, accumulate in abundance and share characteristics with the same metabolites in other plant lineages (Yu M. et al., 2020).

Galloylated catechins are specialized secondary metabolites relatively abundantly present in *C. sinensis* (Wei et al., 2018). Contrary to the small amount of galloylated catechins present in the form of ECG generally in other plants (Bontpart et al., 2018), they are abundant in *C. sinensis*, and EGCG, only massively accumulated in tea plants, is the predominant form (Kim et al., 2004; Steinmann et al., 2013). It is the most bioactive component among the catechin enantiomers, and is derived from the flavonoid branch of the phenylpropanoid metabolite pathway. The acyltransferase family, belonging to subclade 1A of serine carboxypeptidase-like (SCPL) acyltransferases, acts as the most critical downstream gene family involved in the production of EGCG and ECG (Wei et al., 2018). This family extensively expanded to 22 members in the *C. sinensis* genome, while the *Vitis vinifera* genome contains half that number (11) (Wei et al., 2018). Two key enzymes Epicatechin:1-*O*-galloyl-b-*D*-glucose *O*-galloyltransferase (ECGT) and UDP-glucose: galloyl-1-*O*-b-*D*-glucosyltransferase (UGGT) are recruited to catalyze the last two reactions in this bioprocess (Liu et al., 2012). The biosynthesis of galloylated catechins in *C. sinensis* has been comprehensively investigated with regard to the biosynthesis genes of the pathway. However, the transcriptional regulation of these pathways remains to be illuminated.

Genes responsible for plant secondary metabolite biosynthesis are coordinately regulated by TFs, a regulatory superfamily that dynamically drives the evolution of plant metabolic pathways for special compounds (Shoji et al., 2021). The regulatory network of this gene superfamily is highly conserved both in angiosperms and gymnosperms (Zhang et al., 2014). The *R2R3-MYB* TFs confer tissue-specific or development stage-specific patterns for metabolites in the same biosynthesis pathway; often, multiple paralogs coexist in one species (Zhang et al., 2014). Lignin, flavonoids, anthocyanins, and capsaicinoids are four different types of secondary metabolites synthesized from the phenylpropanoid pathway that are regulated by *R2R3-MYB* TFs (Soler et al., 2015; Sun et al., 2016; Zhu et al., 2019). Remarkably, the great expansion of this transcription-regulatory superfamily in plant lineages appears to account for the diversity of regulatory functions that the *R2R3-MYB* TFs undertake in plant-specific metabolic bioprocesses (Millard et al., 2019). As demonstrated in detail by the analysis of Soler et.al, the R2R3-MYB subgroups in *Eucalyptus grandis*, *V. vinifera* and *Populus trichocarpa*, which were equipped with expanded members, greatly determined the diversification of specific functions in lignin biosynthesis (Soler et al., 2015).

Based on the consideration of the massive accumulation of galloylated catechins (ECG and EGCG) in tea plant and tea-specific *R2R3-MYB* TFs identified in this work, we hypothesize that the biosynthesis of the peculiar galloylated catechins is absolutely influenced at the transcription regulation level. Different from the result that concentrates on the involvement of some *CsR2R3-MYB* genes in response to drought, cold, GA, and ABA treatments and proposing a key Cs*R2R3-MYB* gene that regulate some main anthocyanin components, which are revealed in the recent genome-wide report of this family (Chen et al., 2021), we firstly emphasize on confirming the putative *R2R3-MYB* candidates that directly function in the production of *Camellia*-abundantly accumulated compounds (EGCG) among the tea-specific *R2R3-MYB* TFs.

The comprehensive and comparative phylogenetic analysis of *CsR2R3-MYB* TFs, backed by multiple sequence alignment among *C. sinensis* and *A. thaliana*, suggests that most of the members in this family are conserved. Most *R2R3-MYB*s share similar functions to the homologous counterparts studied in *A. thaliana*. Some of the *R2R3-MYB* TFs that clustered in TESA, TESB, and TESC subgroups evolved exclusively in *C. sinensis*, but have isogenous genes in *Actinidia chinensis*, *V. vinifera*, *Theobroma cacao*, and *Coffea canephora* genomes. Thus, we speculated that TESA, TESB, and TESC are either obtained in *C. sinensis* or lost in *A. thaliana* lineages after divergence from their most recent common ancestor during two whole-genome duplication (WGD) events. In addition, members of SMYB5 and S5 subgroups, regulating flavonoids biosynthesis in *A. thaliana*, are greatly expanded in *C. sinensis*, which suggests that they might be either functionally redundant genes or genes that undertake some novel functions in the tea plant.

Considering that galloylated catechins highly accumulate in ABs and young leaves, we speculated that the *CsR2R3-MYB* TFs that were preferentially expressed in these tender tissues along with the major catechin-biosynthesis genes were the most promising candidates putatively regulating the biosynthesis of galloylated catechins. Consistent with previous results (Wei et al., 2018), our study observed high expression levels of key galloylated catechin biosynthesis genes *SCPL1A, ANR, LAR*, and *DFR* in tender tissues, while the expression of upstream genes (*PAL*, *C4H*, and *4CL*) in the phenylpropanoid pathway that are mainly relevant to the generation of condensed polymer PAs, was in fruits, flowers and roots. However, *CsMYB42* was preferentially expressed in tender tissues and had a strong correlation with catechin biosynthesis genes and the contents of ECG and EGCG in the “Shuchazao” variety (Figure 3D), while it was preferentially expressed in old leaves in the “Lingtoudancong” variety (Figure 4C). Thus, differences can be observed in different *C. sinensis* varieties. Eventually, through systematic analyses, *CsMYB30* (TESC subgroup), *CsMYB34* (S5 subgroup), and *CsMYB37* (SMYB5 subgroup) were confirmed as the potential *R2R3-MYB* TFs relevant to the extensive accumulation of ECG and EGCG in *C. sinensis*, however, further investigation is needed. This study has laid a theoretical framework and valuable foundation for the future work, as we have provided a preliminarily evidence for illuminating that the high accumulation of galloylated catechins in *C. sinensis* are likely to be caused by the expanded R2R3-MYB TFs. Nevertheless, it is still necessary to further exploration and validate these results.

## Conclusion

A total of 118 *R2R3-MYB* gene members, classified into 38 subgroups, were identified in the *C. sinensis* genome. Notably, five subgroups (TESA, TESB, TESC, S5, and SMYB5) containing 21 *R2R3-MYB* TFs were identified to be remarkably expanded in or completely unique to *C. sinensis*. Furthermore, gene structure predictions, expression profile validation, and correlation analyses were subsequently conducted to screen out candidate *R2R3-MYB* TFs regulating galloylated catechin biosynthesis. *CsMYB30*, *CsMYB34*, and *CsMYB37* were specifically expanded and their expression level were strongly correlated with galloylated catechin contents, suggesting the probable function in galloylated catechin biosynthesis. The present study firstly revealed the *C. sinensis* specific and expanded R2R3-MYB TFs that are potential regulators of galloylated catechins biosynthesis, which underpinned a basic understanding of the uniquely massive accumulation of galloylated catechins in *C. sinensis.*

## Data Availability Statement

The original contributions presented in the study are included in the article/Supplementary Material, further inquiries can be directed to the corresponding authors.

## Author Contributions

JL performed the qRT-PCR, analyzed and interpreted the data, made the data charts, and wrote the manuscript. SL conceived the project and supervised the research. PC carried out the HPLC test. JC, ST, and WY collected the materials for the experiments and provided useful suggestions. FC reviewed and edited the manuscript. PZ funded the research and reviewed the manuscript. BS designed the project, supervised the research, interpreted the data, and edited the manuscript. All authors contributed to the article and approved the submitted version.

## Conflict of Interest

The authors declare that the research was conducted in the absence of any commercial or financial relationships that could be construed as a potential conflict of interest.

## Publisher’s Note

All claims expressed in this article are solely those of the authors and do not necessarily represent those of their affiliated organizations, or those of the publisher, the editors and the reviewers. Any product that may be evaluated in this article, or claim that may be made by its manufacturer, is not guaranteed or endorsed by the publisher.

## Footnotes

1
https://www.fao.org/faostat/

2
http://tpia.teaplant.org/

3
http://pfam.xfam.org/search/sequence

4
https://www.arabidopsis.org/

5
http://planttfdb.gao-lab.org/

6
https://meme-suite.org/meme/

7
http://weblogo.berkeley.edu/logo.cgi

8
http://bioinformatics.psb.ugent.be/webtools/plantcare/html/

9
https://biodb.swu.edu.cn/qprimerdb/

## Abbreviations

C: catechin
EC: epicatechin
GC: gallocatechin
EGC: epigallocatechin
CG: catechin gallate
ECG: epigallocatechin gallate
GCG: gallocatechin gallate
EGCG: epigallocatechin gallate
qRT-PCR: quantitative reverse transcription polymerase chain reaction
PAL: phenylalanine ammonia lyase
C4H: cinnamate 4-hydroxylase
4CL: 4-coumarate: coenzyme A ligase
CHS: chalcone synthase
CHI: chalcone isomerase
F3H: flavanone-3-hydroxylase
F3 ′ H: flavonoid 3 ′ -hydroxylase
F3 ′ 5 ′ H: flavonoid 3 ′ 5 ′ -hydroxylase
FLS: flavonol synthase
DFR: dihydroflavonol 4-reductase
ANS: anthocyanidin synthase
ANR: anthocyanidin reductase
LAR: leucoanthocyanidin 4-reductase
SCPL1A: subclade 1A of serine carboxypeptidase-like acyltransferases
TFs: transcription factors
TES: tea expanded subgroup
HPLC: high-performance liquid chromatography
Trp/W: tryptophan.
