# Supplementary material for: Systematic Analysis of the R2R3-MYB Family in Camellia sinensis: Evidence for Galloylated Catechins Biosynthesis Regulation
Source: Front Plant Sci. 2022 Jan 3;12:782220. doi: 10.3389/fpls.2021.782220 (PMC8762170; doi:10.3389/fpls.2021.782220)
Supplement: Supplementary file 1 [file Data_Sheet_1.DOCX]

**Systematic analysis of the R2R3-MYB family in Camellia sinensis: evidence for galloylated catechins biosynthesis regulation**

Jingyi Li

Online Supplementary Material


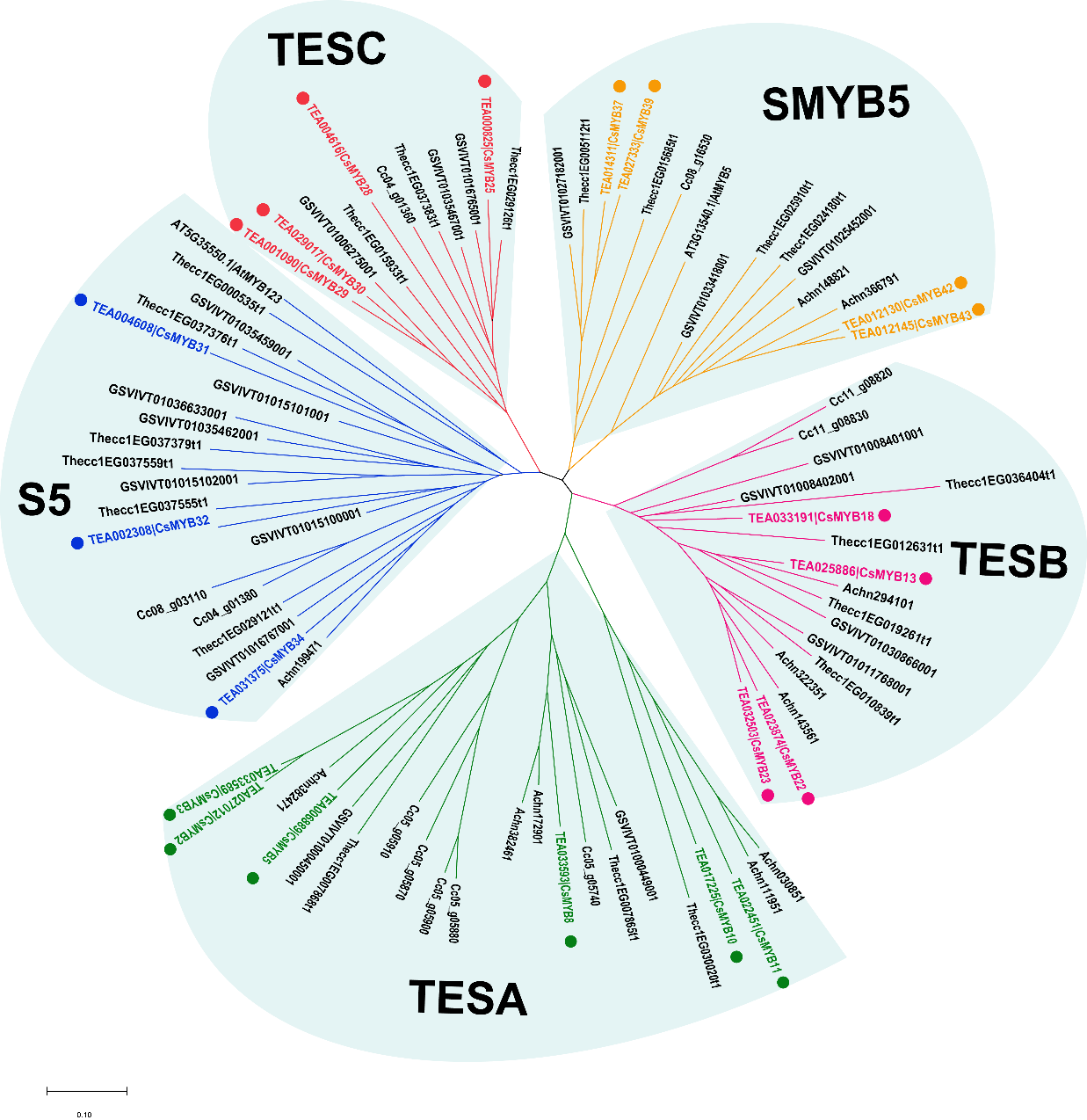


**Supplementary Fig. 1** Phylogenetic analysis of the candidate subgroups in six plant species. A neighbor-joining phylogenetic tree was constructed with the R2R3-MYB proteins from *C. sinensis*, *Actinidia chinensis*, *Vitis vinifera*, *Theobroma cacao*, *Coffea canephora* and *Arabidopsis* genomes. Subgroup short names are indicated beside each clade.


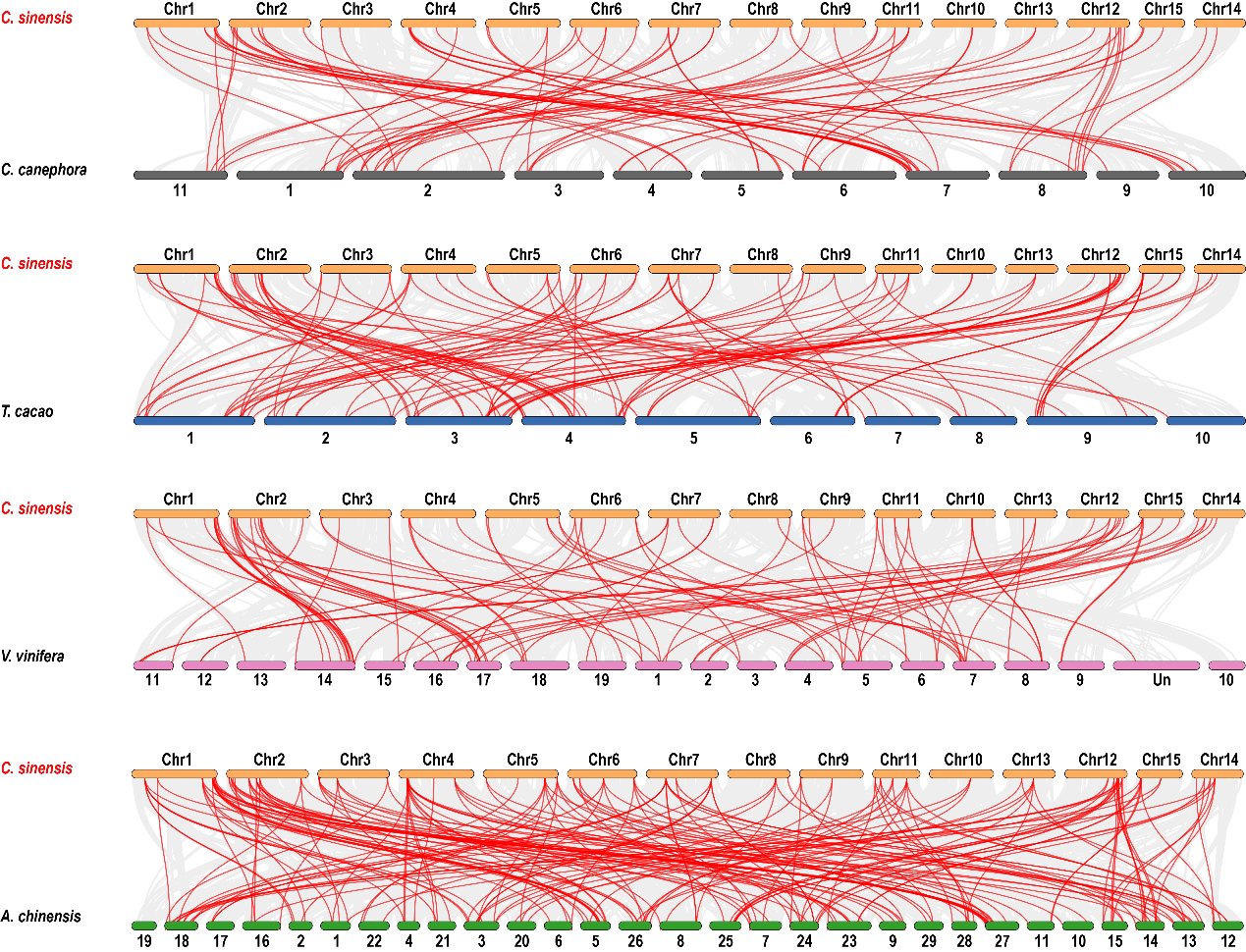


**Supplementary Fig. 2** Synteny analysis of *R2R3-MYB* genes between *C. sinensis* and the four representative plant species (*Coffea canephora, Theobroma cacao, Vitis vinifera, Actinidia chinensis*). Gray lines in the background and red lines represent the collinear blocks and syntenic *R2R3-MYB* gene pairs within *C. sinensis* and other plant genomes, respectively.


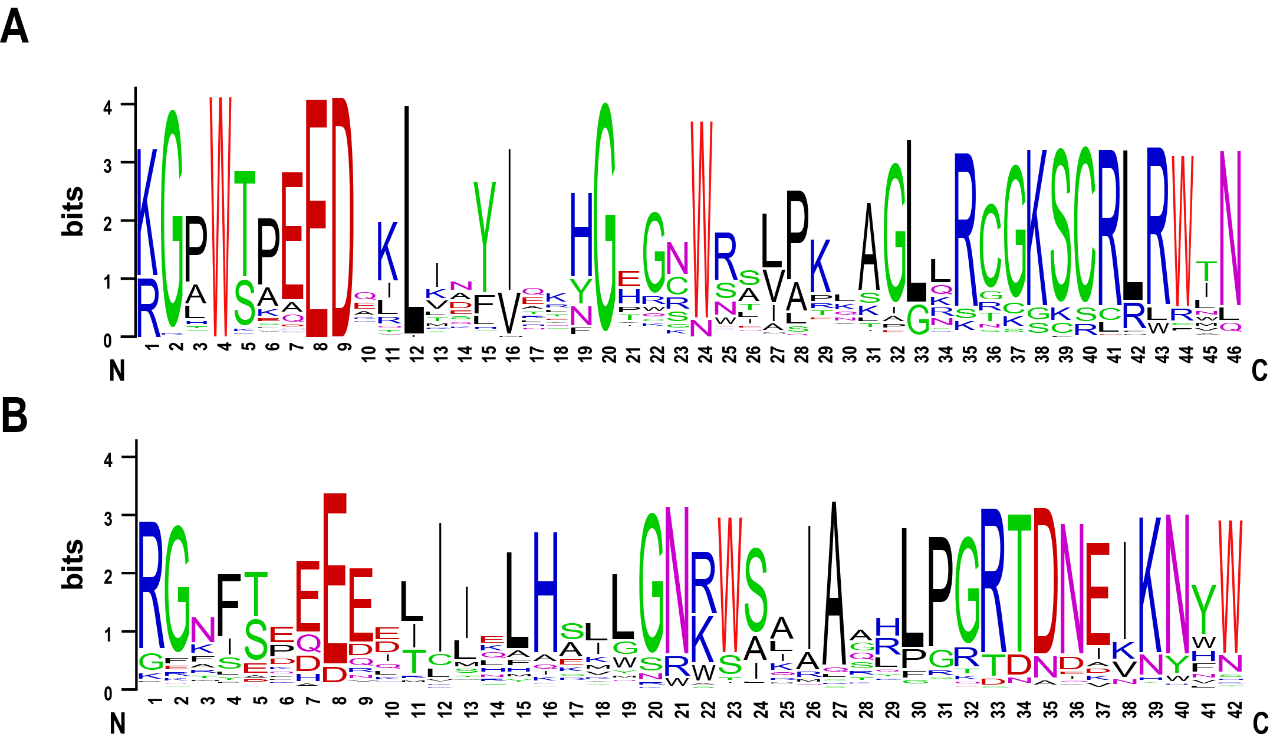


**Supplementary Fig. 3** Analysis of R2 and R3 domains of *C. sinensis* R2R3-MYB TFs. The sequence logos of the R2 (A) and R3 (B) MYB repeats were determined via multiple sequence alignment of the R2R3-MYB proteins. The bit score indicates the information content for each position in the sequence. Highly conserved Trp residues critical for DNA binding in the MYB domain are highlighted in red.


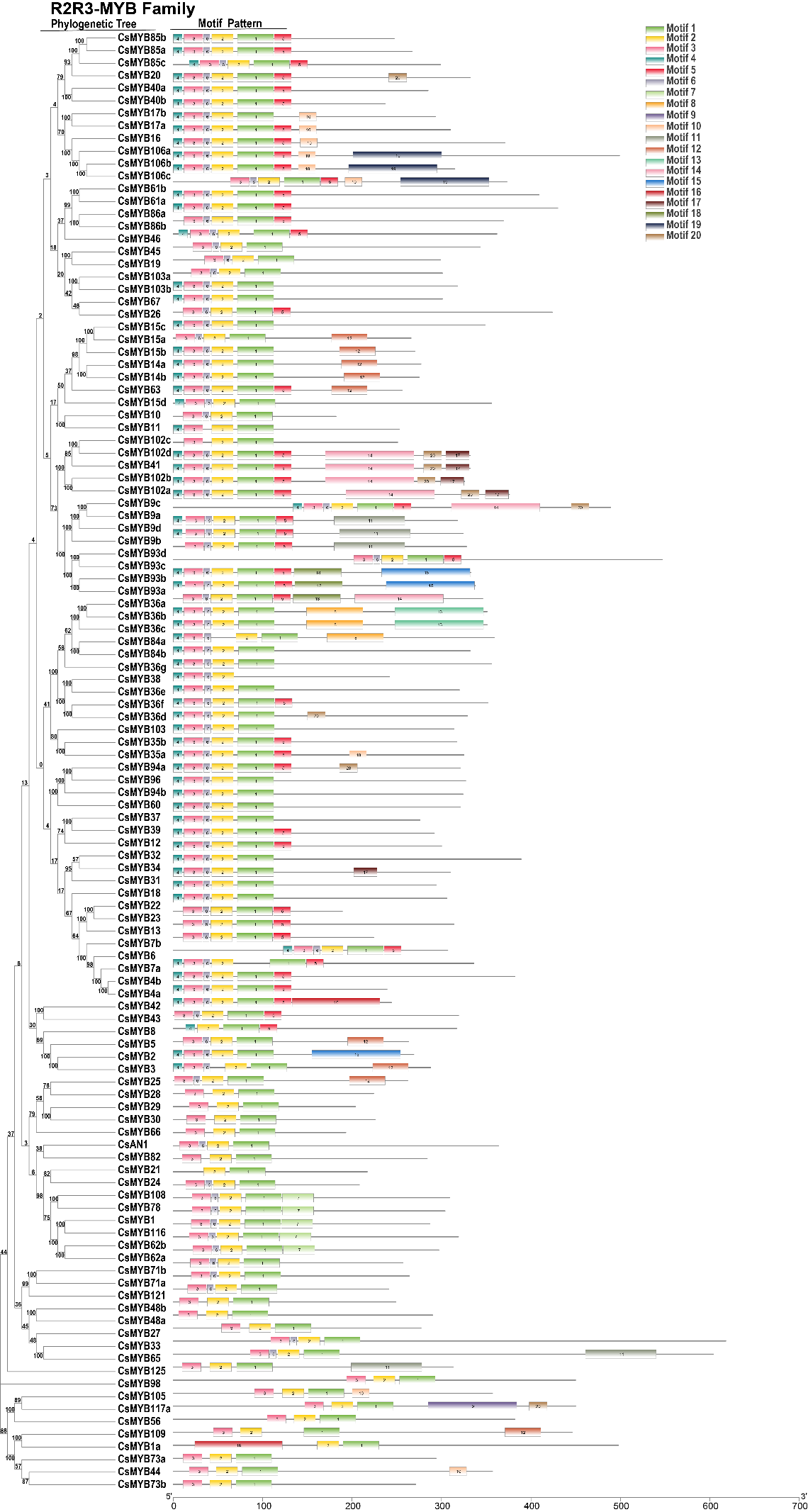


**Supplementary Fig. 4** Phylogenetic relationships and conserved motifs of *C. sinensis* R2R3-MYB TFs. The neighbor-joining tree of 118 R2R3-MYB proteins is shown on the left, and the structures of 20 conserved motifs in R2R3-MYB TFs, predicted by MEME Suite, are shown on the right.


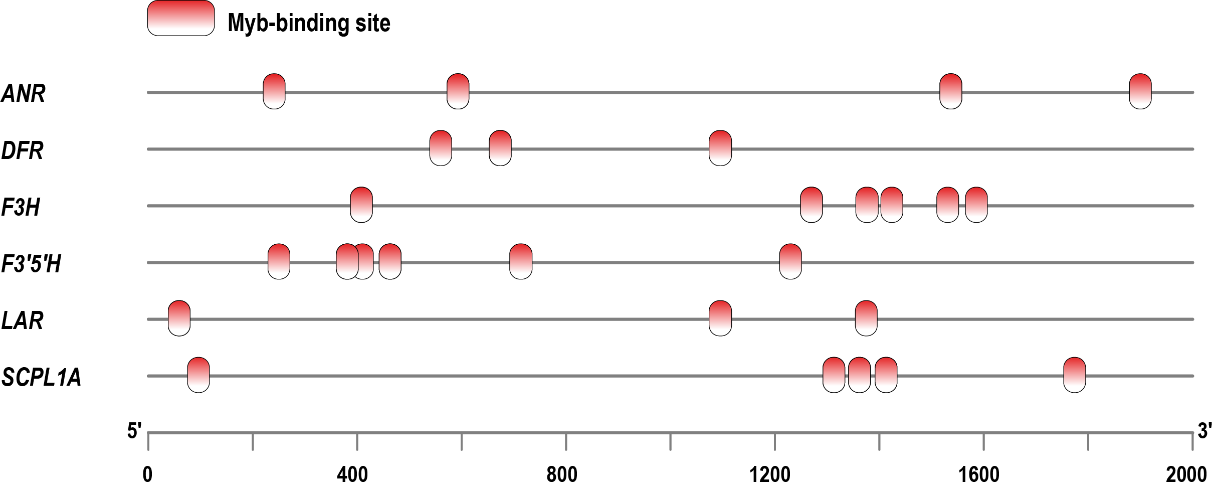


**Supplementary Fig. 5** The Myb-binding sites in the promoters of the catechin biosynthesis downstream genes. Red box represent the Myb-binding site.
